# Paragonimus infection: Rare manifestation with pericardial effusion: A Case report and Literature review

**DOI:** 10.1016/j.idcr.2021.e01075

**Published:** 2021-03-16

**Authors:** Ahmad Al Bishawi, Sarah Salameh, Ahsan Ehtesham, Ihab Massad, Suresh Arachchige, Ahmed Hatim, Issam Bozom, Maliha Thapur

**Affiliations:** a Hamad Medical Corporation, Department of Internal Medicine – Division of Infectious Diseases, Qatar; b Hamad Medical Corporation, Department of Surgery – Division of Cardiothoracic Surgery, Qatar; c Hamad Medical Corporation, Department of Internal Medicine – Division of General Medicine, Qatar; d Hamad Medical Corporation, Department of Clinical Laboratory Medicine and Pathology, Qatar

**Keywords:** MTBc, *Mycobacterial Tuberculosis* complex, Pro-BNP, Pro B-type natriuretic peptide, WBC, white blood cells, RBC, red blood cells, AFB, acid fast bacilli, MTB-PCR, *Mycobacterial Tuberculosis* Polymerase chain reaction, ECG, electrocardiogram, CT, Computed Tomography scan, Paragonimus, Pericardial effusion, Tuberculosis

## Abstract

**Background:**

Paragonimus, is a globally distributed trematode, with human disease limited to endemic regions. It can be transmitted to humans through ingestion of intermediate hosts that are crustaceans. Most symptomatic infections consist of pulmonary disease, and in aberrant migration of immature flukes, extrapulmonary manifestations may occur. These presentations are relatively uncommon and may affect various organs with atypical Clinico-radiological pathologies that are often challenging to diagnose. Pericardial involvement has scarcely been reported before. Furthermore, the management, clinical outcomes and potential complications of this involvement remain unclear.

**Case report:**

Our patient is a 31-year-old Nepalese male who presented with abdominal distension and lower limb oedema. Initial work up revealed pericardial effusion, and analysis was suggestive of exudative lymphocytic effusion. Supported by positive QuantiFERON result along with his demographic data, the patient was treated presumptively as a case of tuberculous pericarditis, despite the negative initial *Mycobacterial Tuberculosis* work up. During follow up, the patient lacked clinical response and repeated echocardiography showed signs of tamponade with concomitant pleural effusion. subsequently video-assisted-thoracoscopy pericardial window along with pericardial and pleural biopsy were performed. Histopathological examination of the biopsied tissue revealed non-necrotizing granulomas containing a parasitic egg suggestive of Paragonimus. Fortunately, the patient received treatment with praziquantel and subsequently made good clinical recovery.

**Conclusion:**

Diagnosis of extrapulmonary Paragonimus infection can be challenging given its rarity and clinical picture mimicking other infectious aetiologies. Pericardial involvement is seldom reported in the literature and clinical suspicion should be raised particularly when dealing with atypical presentations and relevant demographic data.

## Background

Paragonimiasis is a food-borne zoonotic infection caused by one of several *Paragonimus* trematode species also known as lung flukes. The parasite exists in the infective form called metacercariae within aquatic crustaceans such as crabs and crayfish. Upon ingestion of the raw or undercooked seafood, metacercariae excyst in the duodenum and penetrate the intestinal wall to enter the peritoneal cavity. They then migrate to the lungs through the diaphragm wherein they form fibrous cysts and mature into adults [1]. Adult worms begin to deposit eggs that are passed into bronchioles. Rupture of these egg-laden fibrous cysts leads to the late inflammatory manifestation of recurrent hemoptysis. Bronchopleural fistulae can also occur, more commonly with the *Paragonimus skrjabini* than with other species [2]. These species, endemic in China, are also known to cause most of the extra-pulmonary manifestations of the disease [3]. There are approximately 16 species known to cause infection in humans; *Paragonimus westermani* found in the Far East and Southeast Asia is the most common. Additionally, *Paragonimus africanus* in West Africa, *Paragonimus mexicanus* in Central and South America, and *Paragonimus kellicotti* in North America have also been reported to affect humans [4,5]. Although primarily a disease of the lungs, extra-pulmonary involvement does occur, with brain, abdomen and subcutaneous tissue sites being the most common ectopic sites [[6], [7], [8], [9]]. This case represents the uncommon presentation of pericardial involvement, leading to impending tamponade.

## Case report

Our patient is a 31-year-old previously healthy Nepalese gentleman who presented to the emergency department in June 2020 with complaints of abdominal pain and distention associated with bilateral lower limb swelling of about 1-month duration. His abdominal pain was mild to moderate in severity, and not associated initially with nausea, vomiting nor change in his bowel habits. As his symptoms progressed, the patient noticed bilateral lower limb swelling that was present at the time of presentation. Systemic review was unremarkable for fever, weight loss, night sweats nor any new skin rash.

Upon initial assessment, blood pressure was 106/68 mmHg, oxygen saturation of 98 % on ambient air, pulse rate 80 beats per minute and regular. Respiratory rate of 18 breaths per minute. Physical examination revealed muffled heart sounds with raised jugular venous pressure and bilateral lower limb pitting edema. There were no murmurs or added sounds appreciated on cardiac auscultation, and chest exam was clear. The abdomen was mildly distended, with no peritoneal signs and negative for shifting dullness. Systemically, there were no signs of temporal wasting, skin rash, or palpable lymphadenopathy.

Initial labs were significant for a total leukocyte count of 7.6 × 10^3^/ uL with peripheral eosinophilia (9%; absolute eosinophil count 0.7 × 10^3^/uL) other metabolic panels including liver and renal chemistry were within normal limits. Pro B-type natriuretic peptide (Pro-BNP) was elevated with value of 514.80 pg/mL. Basic metabolic workup and cardiac and inflammatory markers were insignificant (Table 1, Table 2). Transthoracic echocardiography was ordered and demonstrated normal global left ventricular function with ejection fraction of 53 % and no regional abnormality, however it also demonstrated the presence of a moderate pericardial effusion. Electrocardiogram (ECG) was ordered which excluded any prior ischemic injury.

**Table 1:** Baseline investigations on subsequent presentation.

	27/06/2020	13/07/2020	Reference Range
**WBC**	7.6 × 10^3^/uL	8.5 × 10^3^/uL	**4.0–10.0**
**Hemoglobin**	13.7 gm/dL	14.5 gm/dL	**13.0–17.0**
**Hematocrit**	42.9 %	44.1 %	**40.0–50.0**
**MCV**	89.4 fl	85.6 fl	**83.0–101.0**
**Platelet**	210 × 10^3^/uL	246 × 10^3^/uL	**150−400**
**Absolute Neutrophil count #**	4.6 × 10^3^/uL	3.5 × 10^3^/uL	**2.0–7.0**
**Lymphocyte #**	1.88 × 10^3^/uL	**3.45** x 10^3^/uL	**1.0–3.0**
**Eosinophil #**	**0.7** x 10^3^/uL	**1.0** x 10^3^/uL	**0.0 – 0.5**
**Neutrophil Percentage**	60.1 %	41.7 %	
**Lymphocyte Percentage**	24.9 %	40.6 %	
**Eosinophil Percentage**	9%	12.0 %

**Table 2:** Blood Chemistry at initial presentation.

	Result	Reference Range		Result	Reference Range
**Urea**	2.99 mmol/L	**3.20** – **7.40**	**Albumin**	32 gm/L	**35.0–50.0**
**Creatinine**	81.0 umol/L	**63.60–110.50**	**ALP**	76.6 U/L	**40.0–150.0**
**Sodium**	136 mmol/L	**135.0–145.0**	**ALT**	43.5 U/L	**0.0–55.0**
**Potassium**	3.7 mmol/L	**3.60 – 5.10**	**AST**	46 U/L	**5.0–34.0**
**Chloride**	103.8 mmol/L	**96.0–110.0**	**NT pro-BNP**	**514.80 pg/mL**	**5.0–74.00**
**Bicarbonate**	25.2 mmol/L	**22.2–29.0**	**Troponin -T**	8.81 ng /L	**0.0–14.00**
**Calcium**	2.09 mmol/L	**2.10 – 2.55**	**Amylase (P)**	17.0 U/L	**8.0–51.0**
**Calcium Corr.**	2.25 mmol/L	**2.10 – 2.55**	**Lipase**	17.0 U/L	**8.0–78.0**
**Bilirubin T**	18.3 umol/L	**3.40 – 20.50**	**Glucose**	5.0 mmol/L	**3.3–5.5**
**Total protein**	62 gm/L	**64.0–83.0**	**CRP**	7 mg/L	**0.0–5.0**

**Fig. 1:**
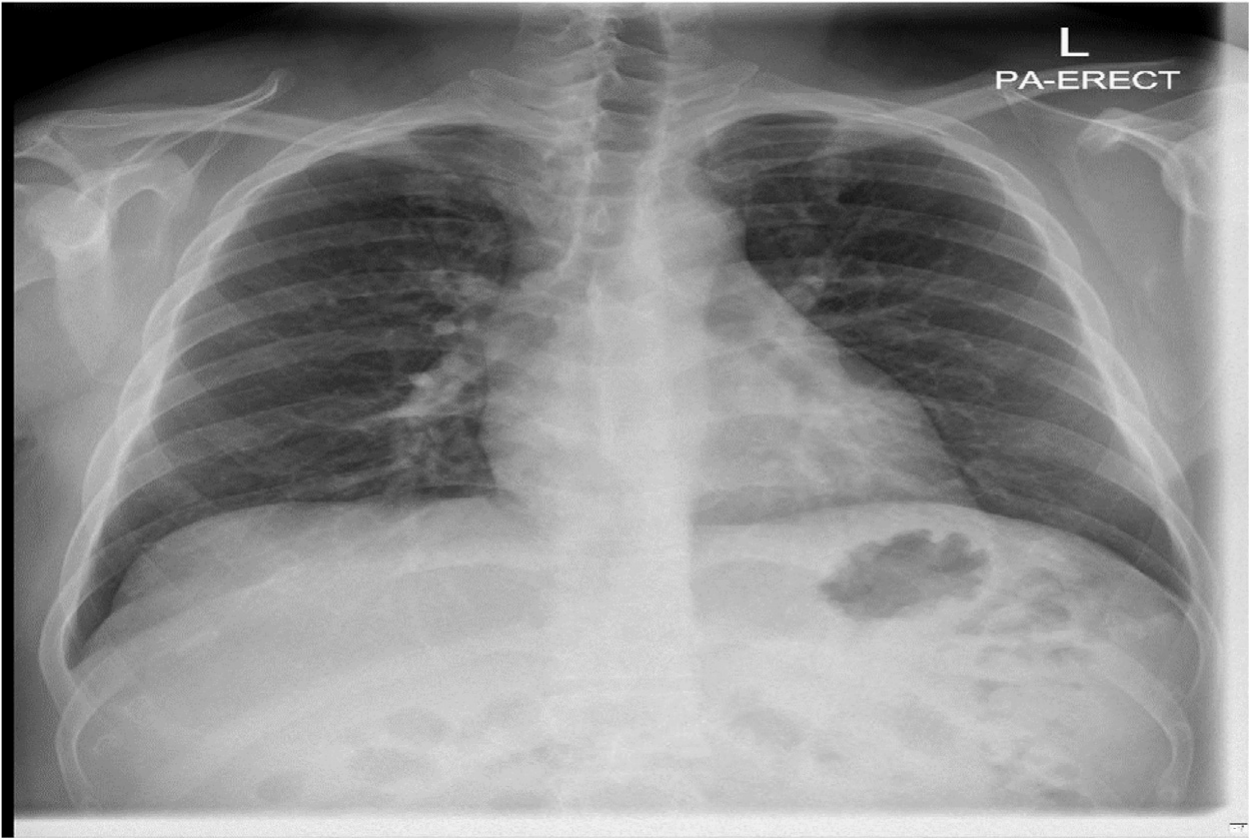
Chest X-ray upon first admission. Cardio-mediastinal silhouette is within normal limits with the heart being normal in size. No pleural effusion / pneumothorax/consolidative patches identified.

Diagnostic pericardiocentesis was performed which showed bloody fluid. Pericardial fluid analysis revealed a White blood cell count (WBC) of 197100 u/L and red blood cells (RBC) of 150 u/L with a predominantly lymphocytic effusion (55 %) (Table 3), Acid fast bacilli (AFB) and *Mycobacterial Tuberculosis complex* (MTBc) -Polymerase Chain Reaction (PCR) from body fluid were negative. MTBc cultures at this point were pending. Ultrasound of abdomen showed only mild ascites. The patient was started on intravenous furosemide. Shortly after admission the patient started complaining of chest pain and increasing shortness of breath. Re-assessment of his vital signs were within normal parameters and repeated examination was unchanged from admission. An urgent ECG did not show any ischemic changes and repeated chest X ray remained clear (Fig. 1); Lab investigations however were significant for an increase in peripheral eosinophilia (12 %; absolute eosinophil count of 1.0 × 10^3^/ uL) (Table 1).

**Table 3:** Body Fluid Hematology.

Pericardial Fluid Analysis			
**Volume BF**	2.0 mL	**WBC BF**	197100U/L
**BF Type Cell Count**	Pericardial	**Neutrophils BF**	15.0U/L
**Color BF**	Bloody	**Lymphocyte BF**	55.0U/L
**Appearance BF**	Bloody	**Monocyte BF**	28.0U/L
**RBC BF**	150U/L	**Eosinophil BF**	2.0U/L

Given the demographic data and pericardial fluid analysis, a clinical suspicion of tuberculosis related pericardial effusion was raised and additional work up with 2 sets of sputum AFB smears, MTB-PCR and MTBc culture were sent along with serum QuantiFERON Gold Plus test. AFB smears and PCR were negative, however QuantiFERON test returned positive. A presumptive diagnosis of pericardial tuberculosis was made, and patient was started empirically on anti-tuberculosis treatment. He received first line regimen with rifampicin, Isoniazid, Pyrazinamide and Ethambutol according to his weight, in addition to prednisolone. He was discharged home to be followed in tuberculosis clinic for the final MTBc cultures.

During clinical follow up the patient was adherent to medications however, still complained of on and off exertional shortness of breath, the final *Mycobacterial Tuberculosis* culture from sputum and pericardial fluid was negative hence repeated echocardiography was requested for re-evaluation.

At 2 months from anti-tuberculosis treatment, repeated echocardiography showed evidence of progressive pericardial effusion with signs of increased intra-pericardial pressure. Echocardiography demonstrated normal global systolic left ventricular function with ejection fraction of 54 % without regional wall motion abnormality but there was circumferential moderate pericardial effusion. There was no right ventricular diastolic collapse or right atrial systolic collapse. The inferior vena cava was borderline in diameter with < 50 % collapse.

**Fig. 2:**
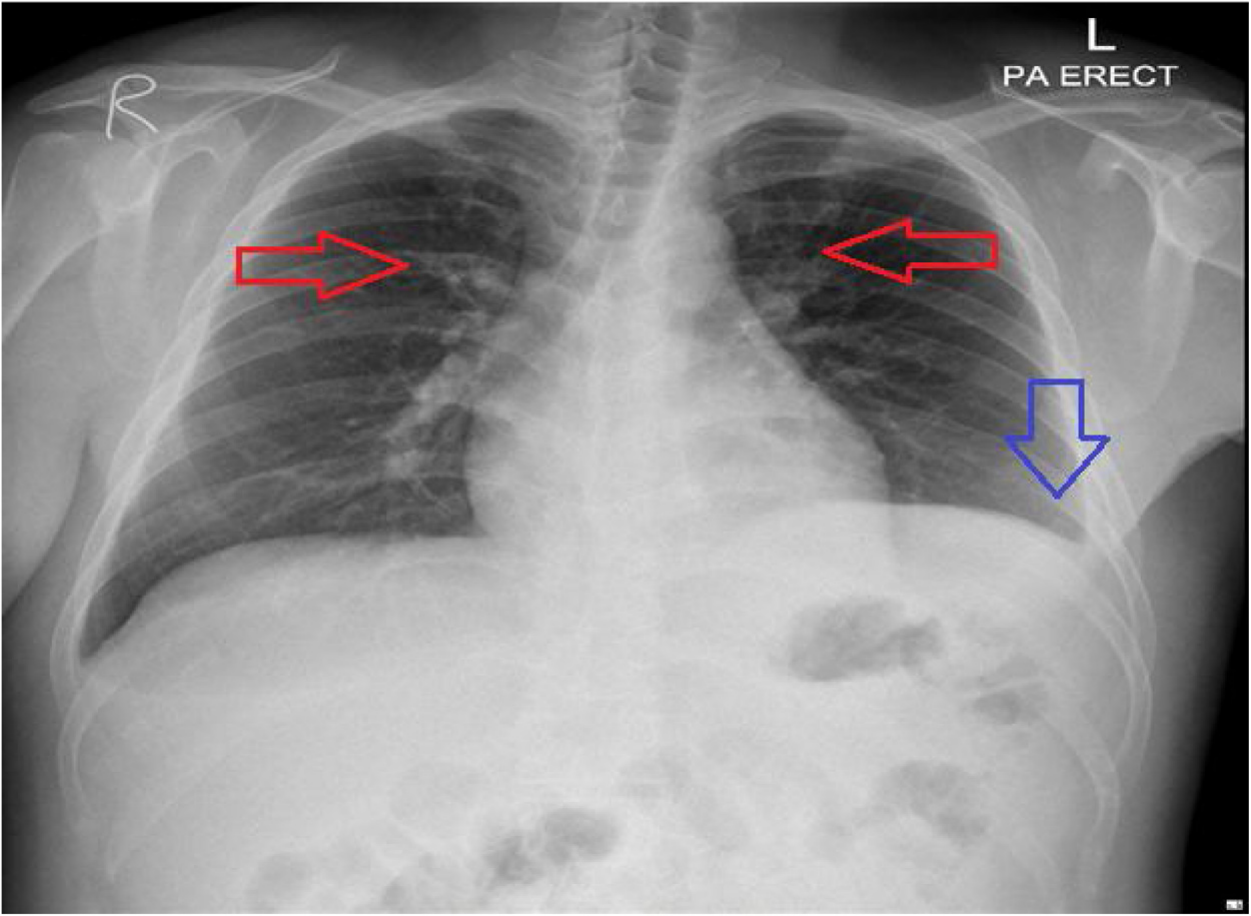
Chest X-ray upon his most recent admission. Mildly prominent hilar vascular markings identified (red arrows) with minimal blunting of the left costophrenic angle (blue arrows) and mild elevation of the left hemidiaphragm. But Cardio-mediastinal silhouette.

**Fig. 3:**
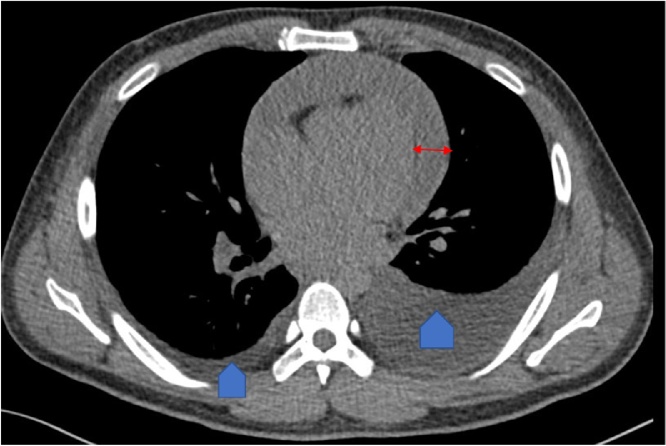
Chest CT upon recent admission. Congestive pulmonary changes in the form of ground glass opacities and pleural effusion at the posterior inferior aspects of both lungs, more on the left. (Arrow heads) Circumferential pericardial effusion (red arrow).

The patient was re-admitted for further evaluation. At the time, examination again demonstrated raised jugular venous pressure with muffled heart sounds on auscultation along and pedal edema. He now had absent breath sounds over the left lung base. Chest x-ray demonstrated new minimal blunting of left costophrenic angle (Fig. 2). Computed Tomography (CT) of the thorax was done and showed bilateral pleural effusion along with congestive pulmonary changes and minimal atelectasis in both lungs and circumferential pericardial effusion of 2.5 cm thickness with no evidence of pericardial calcification (Fig. 3).

**Fig. 4:**
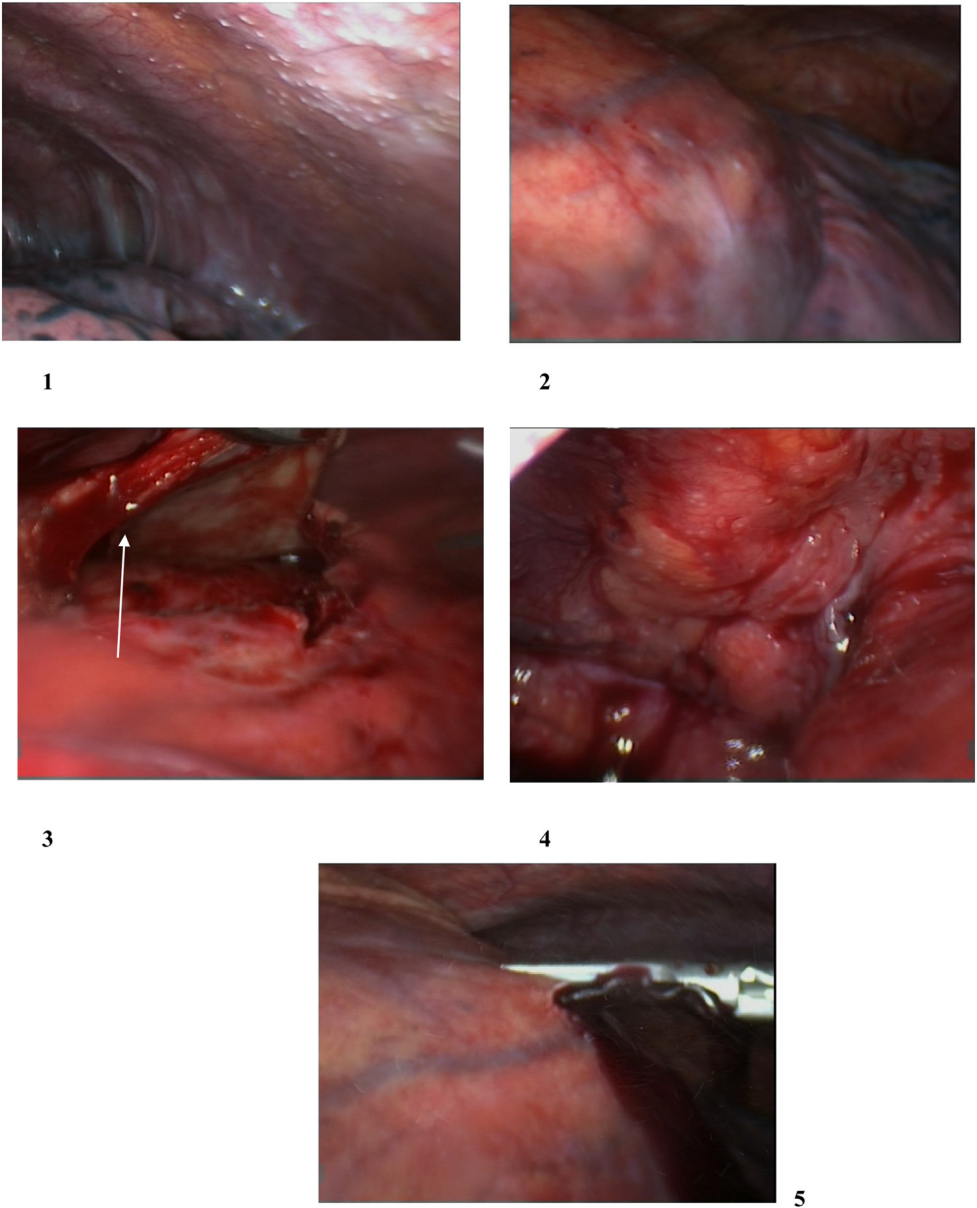
Video-assisted Thoracoscopy findings: 1: Pleural nodules. 2: Pericardial nodules. 3: Thickened pericardium (white arrow). 4: Thickened pleura and pleural nodules at the apex. 5: Sanguineous pericardial effusion pouring out under pressure.

Thoracic surgery team was consulted on the patient and decided to do a thoracoscopic pericardial biopsy and drainage of the pericardial and pleural fluid. A left thoracoscopy was performed and upon inspection of the thoracic cavity multiple white pearly nodules on the parietal pleura and to lesser extent on the pericardium and visceral pleura were found, the pericardium was significantly thickened and inflamed, there were adhesions between the lung and the parietal pleura posteriorly, moderate serous pleural effusion was noted and large bloody pericardial effusion under pressure was relieved upon opening the pericardium (Fig. 4). After creation of the window, biopsies were taken from the pericardial tissue and parietal pleura for histopathology and samples from the pleural and pericardial fluid were sent for microbiology and cytology, tissue cultures were also sent from the pericardium and pleura.

**Fig. 5:**
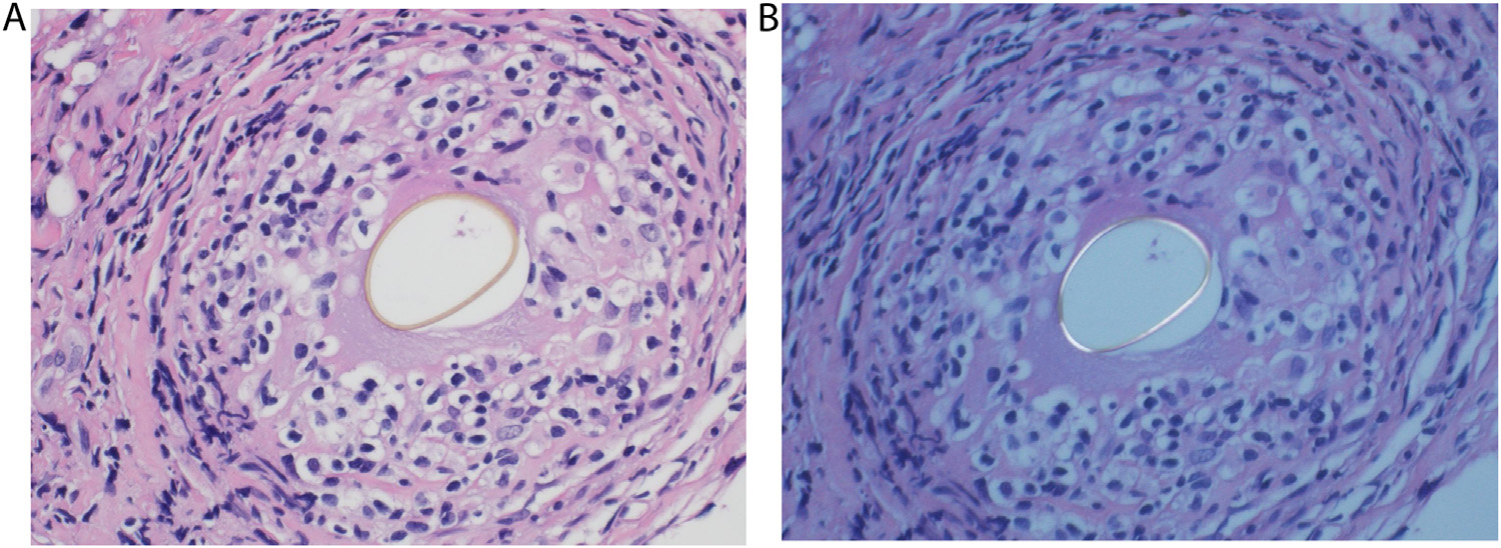
Pleural and Pericardial Biopsy Histopathology report. a. Light microscopic view showing well-defined epithelioid granuloma engulfing parasitic egg (H&E ×400). b. The cuticle of the parasitic egg is polarizable (H&E ×400 with polarizer/analyzer lens).

Ziehl-Neelsen stain for acid fast bacilli and MTBc-PCR from pericardial and pleural tissue as well as fluid in addition to MTBc cultures came back negative. However Microscopic examination of the pleural biopsy revealed thickened and fibrotic pleura which in addition contains clusters and sheets of chronic inflammatory cells including lymphocytes, plasma cells, eosinophils and histiocytes. Few well-defined epithelioid granulomas are seen engulfing parasitic egg with thick cuticle (Fig. 5a) which is polarizable under polarizer/analyzer lens (Fig. 5b) compatible with *Paragonimus Westermani*. Retrospectively, the patient reported eating freshwater crabs in his home country about 2 years earlier and further work up with stool and sputum analysis for ova and parasite was sent but came back negative. Unfortunately, no serological testing could be done for further confirmation even in reference lab in cooperation with our institute.

Anti-tuberculous medications were therefore discontinued, and the patient was started on praziquantel 1800 mg for a total of 3 days (approximately 75 mg/kg in three divided doses) along with a single dose of albendazole 400 mg. Prednisolone was gradually tapered then discontinued. Following treatment patient’s symptoms significantly improved and chest drain was removed after draining a significant amount of fluid. Follow up echocardiography showed almost complete resolution of the effusion and the patient was discharged in good clinical condition.

## Discussion and literature review

Diagnosis of paragonimiasis in this case was delayed given the uncommon presentation of pericardial involvement [[10], [11], [12], [13]] and its context within local epidemiology [14]. Paragonimiasis has rarely been reported in Qatar [15,16] and to our knowledge this is the first such case complicated by constrictive pericarditis.

Clinically, patients may experience a variety of vague often non-specific symptoms according to the lifecycle of the parasite including abdominal symptoms, cough, and fever. Paragonimiasis most commonly presents with cough, hemoptysis and pleural effusion [5]. However, the immature flukes can migrate to tissues other than the lungs and can cause extrapulmonary paragonimiasis. Some of the ectopic sites that have been reported include brain, abdominal organs, female reproductive system and pericardium [6]. Of the several species known, *Paragonimiasis skrjabini,* endemic in China, is known to cause the majority of cases of extrapulmonary involvement of paragonimiasis [21]. One study showed that 11.3 % of patients infected with *P. skrjabini,* presented with cardiac symptoms [7]. There are only a few reported cases of pericardial effusion secondary to Paragonimus infection [[5], [6], [7], [8], [9], [10], [11], [12]].

Diagnosis can be made based on the presence of eggs in the sputum or stool or finding of flukes on biopsy [8]. A study on paragonimiasis in children in southwest China, revealed a large proportion of patients to have elevated leukocytes counts and peripheral eosinophilia. Patients with pericardial effusion comparatively had lower leukocyte counts [9]. In another study on patients diagnosed with paragonimiasis pericardial effusion, positive enzyme-linked immunoassay (ELISA) for *P. skrjabini* was documented in all patients. It also noted the absence of eggs in stool or sputum for *P. skrjabini*. Furthermore, on pathological biopsies of such patients' massive eosinophilic infiltrates were noted [10].

Patients with Paragonimus infection often represent a diagnostic challenge. Pleuropulmonary paragonimiasis is often mistaken for Tuberculosis. This is given the shared epidemiology, presentation of cough, pleuritic chest pain and recurrent hemoptysis [13]. In a study of group infections of Paragonimus, 5 out of 11 patients were initially misdiagnosed as tuberculosis and started on anti-tuberculous treatment for extended durations [14]. Another study conducted in the U.S. found that the median time interval between onset of symptoms to correct diagnosis was 12 weeks (range of 3–83 weeks), with initial diagnoses including pneumonia, bronchitis, influenza, gastroenteritis, acute cholecystitis, and pulmonary embolism [15]. A potentially distinguishing feature from tuberculosis infection, includes an unimpressive fever history, absence of night sweats and weight loss as well as radiologic clues [21]. Chest radiography in pleuropulmonary Paragonimus as opposed to TB usually demonstrate peripheral as well as mid- to lower zone lung lesions as opposed to the apical distribution of tubercular cavities [6]. Pleural fluid analysis can also help guide the differential, with findings of marked eosinophilia, high LDH levels, and low glucose pointing towards a diagnosis of paragonimiasis [20]. In our patient, pericardiocentesis revealed only 2% eosinophils and was probably overlooked as lymphocytic exudative. Diagnosis was finally established by the presence of parasite egg on histopathology in the setting of a non-necrotizing granulomatous inflammation. Lung biopsy and identification of parasitic egg on histopathology was also necessary for the diagnosis of Paragonimus infection in a middle-age immigrant from East Africa similarly presenting with cardiac tamponade despite the negative fluid analysis for tuberculous and fungal cultures [5]. In another case, *Paragonimus westermani* infection was misdiagnosed as aspergillosis in a patient who received anti-fungal treatment before eventually undergoing lobectomy due to adverse effects of the medicine. Pathology revealed presence of Paragonimus and retrospectively, the patient reported history of eating undercooked seafood 3 years back [16]. This highlights the importance of establishing tissue diagnosis in cases with persistent pericardial effusion especially when empiric treatment fails to produce clinically significant response. It also demonstrates the risks associated with treating patients empirically without considering a wide differential for pericarditis.

Fortunately, following the identification of the parasite, the patient was started on the appropriate therapy and anti-tuberculous treatment was stopped. The standard therapy for pulmonary paragonimiasis is 75 mg/kg/day in 3 divided doses for a total of 3 days. This is generally considered adequate treatment for paragonimiasis with high success rate [[17], [18], [19]]. In studies comparing patients with different effusions, pleural, peritoneal and pericardial, patients with pericardial effusions have been observed to require longer hospital stays [9]. Furthermore, recent studies however have demonstrated a need to extend the course of praziquantel in cases of pericardial effusion. In a retrospective study of 57 children with paragonimiasis in China, 54 required at least 3 courses of praziquantel to document satisfactory resolution of pulmonary lesions and concluded that longer course of praziquantel are required in patients with moderate to large pericardial effusions [10]. It is unclear whether this recommendation applies mainly to children or should be extended to adults. Similarly, patients with extensive pleural effusion have been noted to require longer treatment durations [20]. Our patient has received a single course of standard therapy with good clinical and radiologic response. Further follow up will reveal if such treatment is sufficient to prevent future re-accumulation of pleuro-cardiac effusions.

## Conclusion

Diagnosis of paragonimiasis depends on high clinical suspicion with correlation of exposure history, laboratory investigations revealing eosinophilia in the blood and effusive fluids. It also demonstrates the need for early tissue biopsy in order to reveal histopathologic diagnosis in uncommon case presentations. Treatment of Paragonimus pericarditis is successful with a combination of surgical and medical approach.

## Informed consent

Written informed consent was obtained from the patient for publication of this case report and accompanying images

## Declaration of Competing Interest

The authors report no declarations of interest.
